# Miscellany

**Published:** 1904-02

**Authors:** 


### Miscellany.

Retaining Porcelain Inlays.—Erich Schmidt, of Berlin ("The Filling of Teeth with Porcelain," by Walter Wolfgang Bruck, page 49), suggests a novel method of forming the retaining grooves in porcelain inlays. Before filling the matrix with the porcelain paste he places upon the floor of the matrix small pieces of copper wire, and after the inlay is fused he places it into nitric acid, by which the copper is quickly dissolved, leaving grooves well adapted for retention. For inlays fused at a lower temperature than that at which copper melts this will prove an excellent idea, as it enables the operator to form the retaining grooves in positions where they could not be cut with a disk, and of a form more retentive than can be made with a revolving tool. The presence of the copper does not seem to impair the color of the inlay.

---

Asbestos and Alcohol as an Investment.—In some cases alcohol is much to be preferred to water in forming ground asbestos into a paste when it is used as an investment. As soon as the investment is complete, by setting the alcohol on fire, it is dried out quickly, and can be immediately subjected to heat without risk of displacement. When water is used the heat must be applied cautiously, especially in delicate work, to avoid displacement by the water boiling.

---

A Dangerous Feature of Arsenical Paste.—An editorial in the October number of the *Dental Cosmos* (1903) invites attention to several cases in which serious trouble has followed the use of this agent in devitalizing a dental pulp and afterwards filling the tooth temporarily or permanently, without removing the devitalized tissue. In all these cases a mummifying paste had been used. In each case, shortly after the operation the patient had returned to the operator complaining of a continued dull throbbing pain and swelling and tumefaction of the tissues about the socket of the treated tooth, the pain extending deeply into the jaw. This was followed by severe inflammation, exfoliation of the tooth, and much distress during a long period of repair. The writer suggests that this may be due to the arsenic absorbed by the proteid elements of the pulp-tissue and by the hæmoglobin of the vascular supply to the organ being liberated by a breaking down of these tissues and diffused throughout the structure of the root until it sets up an arsenical necrosis in the pericementum. He advises, and urges, great care in the use of this agent, the prompt removal of the devitalized tissue, and a more general use of strictly surgical methods of pulp extirpation under cocaine anæsthesia.

He also suggests that it is highly probable that ingredients of the mummifying paste, when applied to arsenic-saturated pulp-tissue, by virtue of their affinity for proteid matter, upon which their tanning property or their power to bring about the fixation of animal tissue depends, contributes to the result by liberating the arsenic from its original proteid combination in the pulp, thus setting it free to work out its destructive results upon the retentive tissues.

W. H. T.

---

Lead-Pencil Erasing Points as Dental Tools.—Dr. C. H. Land (*Dental Cosmos,* vol xlv., August, 1903, page 617) recommends the rubber erasing points of Dixon's Secretary lead-pencils, No. 2, and his Cabinet, No. 2—724, as useful tools in forming the matrix for inlays. He suggests that the operator be provided with a half-dozen of each, trimmed into suitable sizes and shapes by means of a coarse emory disk in the dental engine. The rubber of which they are made is a soft variety, and especially desirable for this purpose; they are, moreover, purchasable at any book or stationery store, and are inexpensive.

---

A Useful Pain Obtundent.—Chloretone dissolved in chloroform is a convenient pain obtundent in preparing cavities of decay for filling, preparing roots for crowning, and for extracting. Chloretone dissolves sparingly in water, but quite freely in chloroform. A few of the crystals dissolved in a few drops of chloroform forms an effective solution quickly made when needed. A pellet of cotton saturated with this and placed in the cavity for about a minute, makes a very perceptible difference in the pain of excavating. Applied in like manner about a root makes its preparation for crowning far less painful, and assists in extraction. It is also a germicide. It cannot be safely used hypodermically.

---

EXCESS OF SOLDER TO BE AVOIDED IN PORCELAIN WORK.—In an article upon "Porcelain Dental Art" (*Dental Cosmos,* vol. xlv., August, 1903, page 617) Dr. C. H. Land emphasizes the importance of care in avoiding an excess of pure gold used as a solder in constructing platinum jackets and other devices used as a base for porcelain restorations, and, indeed, all work over which porcelain is to be fused. In the first place, an excess is apt to cause discoloration of the porcelain. Secondly, if the platinum is very thin there is danger of the pure gold alloying with it and melting a hole when the work is subjected to the intense heat of the porcelain furnace, just as the base metals will do at a very much lower heat. Thirdly, it is apt to cause warping. Hence it is important to make all joints fit closely and neatly, so as to reduce the amount of pure gold needed as solder to a minimum.

